# Corticosterone Inhibits LPS-Induced NLRP3 Inflammasome Priming in Macrophages by Suppressing Xanthine Oxidase

**DOI:** 10.1155/2020/6959741

**Published:** 2020-05-18

**Authors:** Ling Wu, Chengfu Zhou, Jianfeng Wu, Shikun Chen, Zedan Tian, Quan Du

**Affiliations:** ^1^Department of Anesthesiology, The Second Affiliated Hospital of Chongqing Medical University, Chongqing 400010, China; ^2^Department of Anesthesiology and Perioperative Medicine, Xinqiao Hospital, Army Medical University, Chongqing 400037, China; ^3^State Key Laboratory of Cellular Stress Biology, Xiamen University, Xiamen 361005, China

## Abstract

Following traumatic insult and associated pathogen infection, innate immunity is activated during the perioperative period, especially the NLRP3 inflammasome in macrophages. The neuroendocrine response is also rapidly activated to regulate excessive inflammation; however, the molecular mechanisms are still not completely clear. This study is aimed at investigating the modulation of NLRP3 inflammasome priming by endogenous glucocorticoids (corticosterone, CORT) and its relationship with xanthine oxidase (XO). RAW264.7 murine macrophages were stimulated with LPS (1 *μ*g/ml). LPS-induced NLRP3 expression was pretreated by CORT at different concentrations (0-900 ng/ml). Then, the effect of higher concentrations of CORT (700 ng/ml) on LPS-induced NLRP3 expression and the effect of allopurinol (250 *μ*g/ml) were observed. Finally, the effects of a CORT antagonist (RU486) on XO expression and activity and NLRP3 expression in macrophages were further analyzed. Supernatant levels IL-1*β* and IL-18 were measured. The results showed that LPS-induced NLRP3 expression was upregulated further by pretreatment with CORT (300 ng/ml) (*P* < 0.05); however, higher concentrations of CORT (greater than 700 ng/ml) downregulated NLRP3 expression (*P* < 0.01) and the expression and activity of XO (*P* < 0.05 and *P* < 0.01, respectively). Allopurinol significantly inhibited NLRP3 expression. However, XO expression and activity, NLRP3 expression, and supernatant IL-1*β* and IL-18 levels were significantly increased in the RU486 group compared with the CORT group. In conclusion, our results suggested that CORT inhibits LPS-induced NLRP3 inflammasome priming in macrophages. The underlying mechanism is related to the modulation of XO expression and activity, which may be involved in priming and activating the NLRP3 inflammasome.

## 1. Introduction

An initial traumatic insult disrupts macrobarriers such as the skin, as well as microbarriers such as cell membranes, and constitutes the beginning of a rapidly activated immune response. At the same time, the body's mechanical barriers are destroyed, making it possible for pathogens to invade and amplify a vicious cycle of tissue injury and damaging immunological processes [1–3]. Severe injury may eventually lead to infection-related complications and early multiple organ dysfunction syndrome (MODS). Inflammation is characteristic of activation of innate immunity, which attempts to clear damaged tissues and invading pathogens, with the ultimate goal of maintaining homeostasis. It is crucial for the body to tightly regulate the excessive inflammatory response to maintain balance and homeostasis.

Similar to toll-like receptors (TLRs) of the innate immune system, inflammasomes also orchestrate innate immune responses to aggression, and NOD-, LRR-, and pyrin domain-containing protein 3 (NLRP3) inflammasome is the most studied to date [4]. As a multiprotein complex, the NLRP3 inflammasome is composed of a sensor (NLRP3), an adaptor (ASC), and an effector (pro-caspase-1) [5]. In contrast to transmembrane TLRs localized either to the cell surface or within endosomes [6], NLRP3 is a cytosolic sensor that mostly detects intracellular stimuli. In addition to its critical role in the detection and control of diverse intracellular pathogens [7], NLRP3 also senses and reacts to damage-associated molecular patterns (DAMPs), mediating sterile inflammation after traumatic insult and even some NLRP3 inflammasome-associated diseases. First, pattern recognition or cytokine receptor-induced priming signals lead to the protein synthesis of NLRP3 and pro-IL-1*β*, which are generally required to activate the NLRP3 inflammasome [8]. Then, a second signal promotes NLRP3 inflammasome assembly-mediated cleaved caspase-1 and IL-1*β* secretion. As the central component of the NLRP3 inflammasome, NLRP3 is highly expressed in immune cells, especially macrophages, and its expression is relatively low in resting cells. NLRP3 inflammasome assembly results from the oligomerization of NLRP3, and a priming step must occur first. Thus, NLRP3 inflammasome activity should be effectively controlled, mainly by regulating NLRP3 expression.

The neuroendocrine response is activated as quickly as the innate immunity following trauma and surgery, and its role in regulating immune function has been widely recognized, but the mechanisms are still not completely clear. After traumatic insult or pathogen invasion, the hypothalamic-pituitary-adrenal (HPA) axis is activated, providing important physiological regulation of inflammation through direct and indirect anti-inflammatory effects of adrenal-produced glucocorticoids. As an endogenous glucocorticoid in rodents, corticosterone (CORT) is the major end product of the activated HPA axis after stress. Glucocorticoids have well-known anti-inflammatory effects and are still widely used to treat many inflammatory diseases, but the exact mechanism is unclear. It is possible for the body to regulate inflammation by modulating the NLRP3 inflammasome via CORT.

Surgical injury not only leads to tissue injury but also destroys the body's barriers and thus exposes the innate immune system to a variety of DAMPs and pathogen-associated molecular patterns (PAMPs). In fact, these triggers for NLRP3 inflammasome activation are heterogeneous [9]. Notably, reactive oxygen species (ROS) generation is often shared by multiple pathways to regulate inflammasome activation [10]. When ROS production is suppressed by scavengers, NLRP3-induced IL-1*β* generation is impaired [11]. However, macrophages derived from mice in which the NADPH oxidase (NOX1, NOX2, or NOX4) components of the NOX complex were knockout did not have impaired IL-1*β* secretion [12]. Mitochondrial ROS may have an alternative intracellular source, and there are data linking mitochondrial stress to ROS production [13]. In fact, many exogenous and endogenous factors promote NLRP3 expression, including TLR agonists, proinflammatory cytokines, and ROS. Moreover, a recent study demonstrated that xanthine oxidase- (XO-) derived mitochondrial ROS was the trigger for NLRP3 expression and finally its inflammasome activation [14].

Given the critical role of the NLRP3 inflammasome in the innate immune response, the regulation of NLRP3 expression and its inflammasome priming may be involved in countering excessive inflammatory responses. Therefore, we hypothesized that the endogenous glucocorticoid CORT, released in large quantities after surgical stress, modulates NLRP3 inflammasome activity through the regulation of XO. As a model system, RAW264.7 cells were stimulated with LPS to induce NLRP3 expression and prime the NLRP3 inflammasome. In the present study, we investigated the role of CORT in the modulation of LPS-induced NLRP3 inflammasome priming and its relationship with XO and offer new insights for the modulation of innate immunity to maintain homeostasis by endogenous glucocorticoids after surgical injury.

## 2. Methods

### 2.1. Cell Culture

The murine macrophage cell line RAW264.7 was obtained from ATCC and maintained in DMEM (Gibco, USA) supplemented with 10% fetal bovine serum (HyClone), 100 U/ml penicillin, and 100 *μ*g/ml streptomycin. Cells were cultured in a 5% CO_2_ incubator at 37°C and were used for experiments until the cell growth density was approximately 80%. The ASC-reconstituted RAW264.7 cell line was from the State Key Laboratory of Cellular Stress Biology (Xiamen University, China).

### 2.2. Preparation of Stock Solutions

LPS (Sigma, St Louis, MO) was diluted to a concentration of 1 mg/ml in PBS as a stock solution and stored at -20°C until use. CORT (Sigma, St Louis, MO) was diluted to a concentration of 1 mg/ml in DMSO as a stock solution and stored at -80°C until use. The stock solution of CORT was diluted to different final concentrations in PBS and then used. Allopurinol (Sigma) was diluted to a concentration of 10 mg/ml, and RU486 (MCE) was diluted to a concentration of 1 *μ*M in DMSO as a stock solution.

### 2.3. Inhibition and Stimulation of Cells

Cells were primed with 1 *μ*g/ml LPS for 2 h, unless stated otherwise. After pretreatment with different concentrations of CORT (0-900 ng/ml) for 1 h, the cells were stimulated with LPS for an additional 1 h. To examine the effect of XO, after pretreatment with allopurinol (250 *μ*g/ml) for 1 h, the cells were stimulated further with LPS. Pretreatment with RU486, an antagonist of CORT, was performed. Supernatants were collected, the cells harvested, and protein, RNA, and enzyme activity were analyzed.

### 2.4. Western Blot Analysis

Cells were lysed in RIPA lysis buffer. Total cell protein was extracted and quantified. Blots were prepared using polyacrylamide gels and nitrocellulose membranes using a Bio-Rad II system. The antibodies used were rabbit anti-mouse NLRP3 (CST), rabbit anti-mouse caspase-1 (ImmunoWay), rabbit anti-mouse XO (Abcam), rabbit anti-mouse GAPDH (Bioworld), and goat anti-rabbit conjugated to horseradish peroxidase-2 (Zhongshan Biological Company, Beijing, China). The images were obtained and analyzed by using an ECL developer and gel image analysis system.

### 2.5. PCR and Quantitative Real-Time PCR

Briefly, cells were placed into Trizol (Takara), and RNA was extracted and reverse transcribed into cDNA using a kit (Takara). Relative expression levels of RNA transcripts were determined using gene-specific primers, SYBR green, and a PCR instrument (Bio-Rad).

The sequences of the primers used were as follows: mouse NLRP3 sense 5′-CCAGACCTCCAAGACCACTACG-3′ and antisense 5′-GCTTCCGCAGATCACACTCCT-3′; mouse XO sense 5′-TATGGGGTGGCTTGCTCAGA-3′ and antisense 5′-CAAGACCCTGGACAAATGCC-3′; mouse GAPDH sense 5′-GACATCAAGAAGGTGGTGAAGC-3′ and antisense 5′-GAAGGTGGAAGAGTGGGAGTT-3′.

### 2.6. XO Activity

XO activity was measured according to the manufacturer's protocol (Solarbio, China). Cells were collected and precipitated, and the reaction fluid was added. The cells were lysed by ultrasound (200 W), and then, the supernatant was separated by centrifugation (4°C, 8000 rpm, 10 min). After the supernatant (500 *μ*l) was aspirated, the working fluid was added and mixed. The initial absorbance value (A1) at 290 nm and the absorbance value after 1 minute (A2) were read and recorded using a microplate reader (BioTek, USA). The activity was calculated by using the formula XO (nmol/min) = 8.52 × (A2 − A1).

### 2.7. ELISA

Supernatants from macrophages were transferred into the plate containing enzyme-linked antibodies against IL-1*β* and IL-18. After color development, the absorbance at 450 nm was detected by an Infinite 2000 (TECAN, Grodig, Austria).

### 2.8. Graphical Representation and Statistical Analysis

The results are expressed as mean (s.d.) of the data obtained from at least three experiments. Comparisons among groups were made using one-way analysis of variance (ANOVA), and significance was set at *P* < 0.05. Figures and statistics were generated by GraphPad Prism version 6 and SPSS11.0.

## 3. Results

### 3.1. LPS Induces NLRP3 Expression and NLRP3 Inflammasome Priming in Macrophages after Stimulation for Various Periods of Time

**Figure 1:**
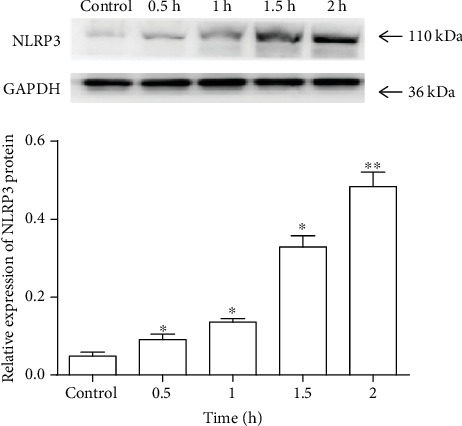
Expression of NLRP3 protein in RAW264.7 cells treated with LPS (1 *μ*g/ml) for different times. NLRP3 protein expression was analyzed by Western blotting. In the control group, cells were stimulated with LPS at 0 h. Values are mean (s.d.) (*n* = 5). ∗*P* < 0.05, ∗∗*P* < 0.01 versus control.

A sufficient level of NLRP3 protein is necessary to form and activate the NLRP3 inflammasome, and its priming is typically accomplished in vitro using a microbial TLR. In the present study, RAW264.7 mouse macrophages were stimulated with LPS. NLRP3 protein expression in resting macrophages was relatively low in the control group, and assembly of the NLRP3 inflammasome complex was poorly induced. NLRP3 expression at the protein level was significantly increased after stimulation by LPS from 0.5 h to 1.5 h in comparison with that of the control (*P* < 0.05), reaching the highest level at 2 h (*P* < 0.01) (Figure 1). NLRP3 mRNA was also increased significantly compared with that of the control (Fig. S1) (supporting information). These results indicate that in our model system, LPS enhances NLRP3 expression consistently, leading to NLRP3 inflammasome priming in macrophages.

### 3.2. CORT Modulates NLRP3 and Cleaved Caspase-1 Expression in a Concentration-Dependent Manner

**Figure 2:**
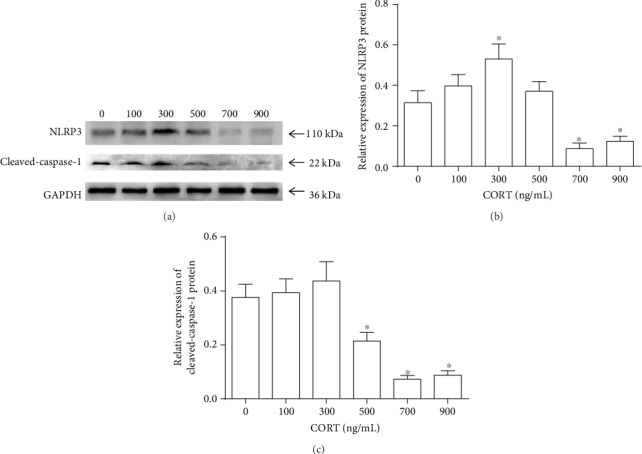
LPS-induced protein levels of NLRP3 and cleaved caspase-1 expression in RAW264.7 cells pretreated with different concentrations of CORT. After pretreatment with different concentrations of CORT for 1 h, the cells were stimulated with LPS (1 *μ*g/ml) for an additional 1 h. (a) Western blots showing NLRP3 and cleaved caspase-1 expression. (b) Statistical chart showing the relative expression of NLRP3 protein. (c) Statistical chart showing the relative expression of cleaved caspase-1 protein. Values are mean (s.d.) (*n* = 5). ∗*P* < 0.05, ∗∗*P* < 0.01 versus control.

CORT is the major endogenous glucocorticoid in rodents. In addition to the effect on NLRP3 expression in macrophages induced by LPS, different concentrations of CORT may modulate NLRP3 expression after stress. To examine whether CORT influences NLRP3 expression in macrophages, we added increasing concentrations of CORT (0 to 900 ng/ml) to the cells. NLRP3 protein expression was observed in control macrophages treated with only LPS without CORT (0 ng/ml). Pretreatment with low concentrations of CORT (100 ng/ml) slightly increased NLRP3 protein expression. A concentration of 300 ng/ml CORT significantly increased NLRP3 protein expression compared with that of the control (*P* < 0.05) (Figures 2(a) and 2(b)). In contrast, NLRP3 protein expression was significantly decreased after exposure to high concentrations of CORT (700 ng/ml and 900 ng/ml), but not at 500 ng/ml (Figures 2(a) and 2(b)). During NLRP3 inflammasome priming, pro-caspase-1 is cleaved, promoting NLRP3 inflammasome activation. Additionally, CORT also modulated cleaved caspase-1 expression similar to its effect on NLRP3 expression (Figures 2(a) and 2(c)), indicating that CORT might modulate NLRP3 inflammasome priming and its final activation. Together, these results suggest that CORT modulates NLRP3 expression and inflammasome priming, which is concentration dependent.

### 3.3. Higher Concentrations of CORT Downregulate NLRP3 and XO Expression and Suppress XO Activity

**Figure 3:**
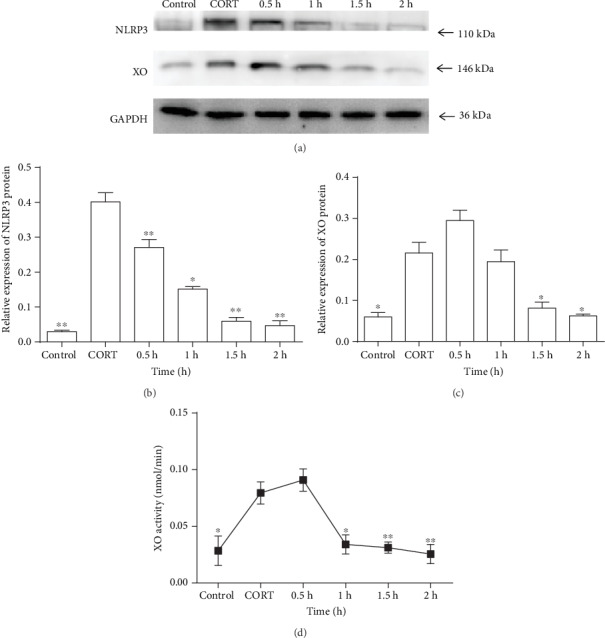
Protein levels of NLRP3 and XO and XO activity in RAW264.7 cells treated with CORT and LPS. After pretreatment with 700 ng/ml CORT for 1 h, the cells were stimulated with LPS (1 *μ*g/ml) for different times. In the control group, there were no treatments. In the CORT group, pretreatment with 700 ng/ml of CORT for 1 h was followed by stimulation with LPS at 0 h. (a) Western blots showing NLRP3 and XO expressions. (b) Statistical chart showing the relative expression of NLRP3 protein. Statistical charts showing the (c) relative expression of XO protein and (d) XO activity. Values are mean (s.d.) (*n* = 6). ∗*P* < 0.05, ∗∗*P* < 0.01 vs. the CORT group.

In the control group, NLRP3 protein expression was low in the absence of treatment with LPS and CORT. In the CORT group, NLRP3 expression was increased after pretreatment with CORT without LPS stimulation (*P* < 0.01) (Figures 3(a) and 3(b)), indicating that CORT directly modulates and primes innate immunity. We chose 700 ng/ml as a higher concentration of CORT after stress. In addition to TLR agonists, XO-derived ROS production might be involved in NLRP3 expression. We measured XO expression in macrophages in the presence of LPS stimulation and the higher concentration of CORT (700 ng/ml). Consistent with the above results, CORT significantly suppressed NLRP3 protein expression in cells stimulated by LPS for 0.5 h to 2 h in comparison with that of the CORT group (i.e., LPS stimulation was performed at 0 h after CORT (700 ng/ml) pretreatment for 1 h) (*P* < 0.01 or *P* < 0.05) (Figures 3(a) and 3(b)); CORT also downregulated NLRP3 mRNA expression (*P* < 0.01) (Fig. S2). Moreover, XO protein expression was downregulated gradually by CORT in comparison with that of the CORT group and was significant with LPS stimulation (1 *μ*g/ml) for 1.5 h to 2 h (*P* < 0.05) (Figures 3(a) and 3(c)). Additionally, XO mRNA level was also downregulated (Fig. S3). XO protein expression is the major determinant of XO activity. Furthermore, compared with XO activity in the CORT group, CORT also suppressed XO activity from 1 h to 2 h (*P* < 0.05 or *P* < 0.01) (Figure 3(d)). Taken together, these results indicate that higher concentrations of CORT downregulate not only NLRP3 expression but also XO expression and its activity, which indicates a close link between XO activity and the NLRP3 inflammasome.

### 3.4. XO Regulates NLRP3 Expression at the mRNA and Protein Levels

**Figure 4:**
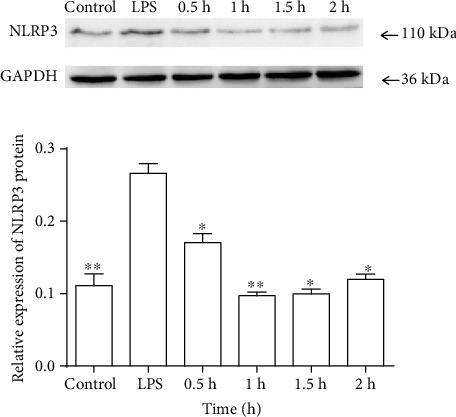
NLRP3 protein expression in RAW264.7 cells treated with allopurinol. After pretreatment with 250 *μ*g/ml of allopurinol for 1 h, the cells were stimulated with LPS (1 *μ*g/ml) for different times. In the control group, there was no treatment. In the LPS group, the time point for stimulation with LPS was 2 h with no allopurinol pretreatment. Values are mean (s.d.) (*n* = 6). ∗*P* < 0.05, ∗∗*P* < 0.01 versus the LPS group.

Allopurinol is an XO inhibitor. To demonstrate that XO regulates NLRP3 expression, allopurinol (250 *μ*g/ml) was used. Following pretreatment with allopurinol, LPS-induced NLRP3 protein expression was downregulated after stimulation with LPS from 0.5 h to 2 h (*P* < 0.05 or *P* < 0.01) (Figure 4); NLRP3 mRNA was also inhibited significantly from 0.5 h to 2 h (Fig. S4). Taken together, these results suggest that XO regulates LPS-induced NLRP3 expression in macrophages.

### 3.5. CORT Inhibits NLRP3 Expression via Suppression of XO

**Figure 5:**
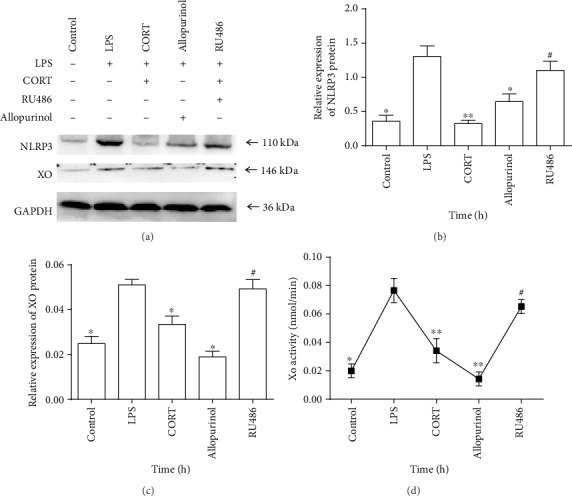
Protein levels of NLRP3 and XO and XO activity in RAW264.7 cells treated with different combinations of LPS, CORT, allopurinol, and RU486. The control group received no treatment. In the LPS group, cells were stimulated with LPS (1 *μ*g/ml) for 2 h. Cells were pretreated with CORT (700 ng/ml) for 1 h and stimulated with LPS (1 *μ*g/ml) for 2 h in the CORT group. In the allopurinol group, cells were pretreated with allopurinol (250 *μ*g/ml) for 1 h and stimulated with LPS (1 *μ*g/ml) for 2 h. Cells were pretreated with RU486 (1 *μ*M) for 1 h, CORT (700 ng/ml) for additional 1 h, and then LPS (1 *μ*g/ml) for 2 h in the RU486 group. (a) Western blots showing NLRP3 and XO expressions. (b) Statistical chart showing relative NLRP3 expression. Statistical charts showing (c) relative XO expression and (d) XO activity. Values are mean (s.d.) (*n* = 5). ∗*P* < 0.05, ∗∗*P* < 0.01 versus the LPS group; ^#^*P* < 0.05 versus the CORT group.

RU486 is an antagonist to glucocorticoids receptor (GR). Because of the role of XO in regulating NLRP3 expression, RU486 was used to investigate whether CORT inhibits NLRP3 expression by suppressing XO. In the different drug combination experiments, the protein levels of NLRP3 and XO and XO activity were all lower in the control group compared with those of the LPS group (*P* < 0.05) (Figures 5(a)–5(d)), indicating that NLRP3 and XO expression is primed by LPS. When allopurinol was administered to inhibit XO before LPS stimulation, LPS-induced XO and NLRP3 expression were both attenuated (*P* < 0.05). In the CORT group, NLRP3 and XO expressions were downregulated (*P* < 0.01 and *P* < 0.05, respectively). Finally, pretreatment with RU486 increased NLRP3 protein expression (*P* < 0.05) and enhanced XO protein expression and XO activity in comparison with those of the CORT group (*P* < 0.05) (Figures 5(a)–5(d)). Taken together, these results suggest that CORT inhibits LPS-induced NLRP3 expression by suppressing XO in macrophages.

### 3.6. CORT Inhibits LPS-Induced NLRP3 Inflammasome Priming and Activation in Macrophages by Suppression of XO

**Figure 6:**
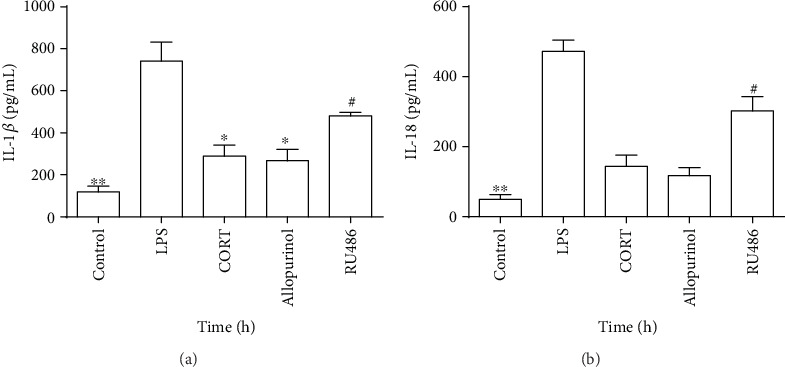
Supernatant levels of IL-1*β* and IL-18 in ASC-reconstituted RAW264.7 cells treated with the combination of different drugs. The control group received no treatment. In the LPS group, cells were stimulated with LPS (1 *μ*g/ml) for 2 h. In the CORT group, cells were pretreated with CORT (700 ng/ml) for 1 h and stimulated with LPS (1 *μ*g/ml) for 2 h. In the allopurinol group, cells were pretreated with allopurinol (250 *μ*g/ml) for 1 h and stimulated with LPS (1 *μ*g/ml) for 2 h. In the CORT group, cells were pretreated with RU486 (1 *μ*M) for 1 h, then treated with CORT (700 ng/ml) for an additional 1 h, and were finally stimulated with LPS (1 *μ*g/ml) for 2 h. After the cells were pretreated with different drugs and primed with LPS, ATP was applied for 1 h in all groups. (a) Supernatant levels of IL-1*β*. (b) Supernatant levels of IL-18. Values are mean (s.d.) (*n* = 5). ∗*P* < 0.05, ∗∗*P* < 0.01 versus the LPS group; ^#^*P* < 0.05 versus the CORT group.

The RAW264.7 macrophage cell line does not express the ASC gene, but the cells resemble bone marrow-derived macrophages (BMDMs) when ASC is ectopically introduced [15]. In this study, the ASC-reconstituted RAW264.7 cell line was used to investigate inflammasome activation. Supernatant levels of IL-1*β* and IL-18 were both lower in the control group compared to those of the LPS group (*P* < 0.01) (Figures 6(a) and 6(b)), indicating that the NLRP3 inflammasome was activated by LPS plus ATP. Allopurinol decreased the supernatant level of IL-1*β* (*P* < 0.05). In the CORT group, supernatant IL-1*β* levels were also decreased (*P* < 0.05). Finally, pretreatment with RU486 increased the levels of both IL-1*β* and IL-18 (*P* < 0.05) (Figures 6(a) and 6(b)). Overall, these results confirm that LPS induces NLRP3 inflammasome priming by regulating XO and activates the NLRP3 inflammasome in macrophages as indicated by increased supernatant IL-1*β* and IL-18 levels and these effects are inhibited by CORT.

## 4. Discussion

This study showed that endogenous glucocorticoid CORT modulated NLRP3 expression and its inflammasome priming in macrophages to regulate inflammation, and higher concentrations of CORT suppressed LPS-induced NLRP3 expression and its inflammasome activity, which was closely related to XO.

Surgical injury leads to endogenous molecules and possible pathogen invasion, which are sensed by the common pattern recognition receptors (PRRs) and induce a series of proinflammatory immune responses [16, 17]. In addition to the severe systemic inflammatory response following major surgery or trauma, deaths are often caused by infections and septic complications during the perioperative period. Similar to TLRs, the NLRP3 inflammasome has also been shown to play a critical role in innate immune responses to injury and infection [4]. A wide range of endogenous DAMPs and exogenous PAMPs can trigger NLRP3 inflammasome activation, making it the perfect candidate for inflammasome-mediated responses during surgical injury and associated infection [18]. NLRP3 inflammasome activation results in the secretion of proinflammatory cytokines, including IL-1*β* and IL-18, contributing to the systemic inflammatory response [19, 20]. Interestingly, it has been shown that inflammasome activation also plays a critical role in the passive and active extracellular release of high-mobility group box 1 (HMGB1), a well-characterized DAMP [21]. In addition to inflammatory mediator production, NLRP3 inflammasome activation leads to pyroptotic cell death, resulting in the release of intracellular contents into the extracellular space, contributing to enhancing and propagating “sterile” inflammation [22]. Moreover, the NLRP3 inflammasome is also involved in the response to diverse pathogens, including amoeba, protozoans, fungi, bacteria, and viruses [23].

Despite research on the NLRP3 inflammasome, the regulation of NLRP3 inflammasome activity is still not well understood. The protein level of NLRP3 is critical for inflammasome assembly and is regarded as a rate-limiting element for inflammasome activation [24], demonstrating that the level of NLRP3 expression determines its activity [25]. Of interest, in contrast to murine monocytes, strong LPS priming signals alone are sufficient to trigger NLRP3 inflammasome activation in human monocytes in some conditions, although activation signals may remain necessary for potentiation of weak priming signals [26, 27]. These differential requirements may be due to endogenous ATP secretion by human monocytes upon priming. Released ATP may then act as an activation signal in an autocrine/paracrine manner [27]. In the present study, LPS consistently increased NLRP3 expression in RAW264.7 mouse macrophages, reaching the highest level at 2 h. Consistent with previous reports [15, 28], in our model system, LPS induced NLRP3 expression and priming in macrophages.

Glucocorticoids are the central effector hormones for the HPA axis, which is responsible for maintaining homeostasis in response to stress. A growing body of evidence suggests that, far from being suppressive, endogenous glucocorticoids might enhance immune function to show a proinflammatory effect and defend against further injury or pathogens, especially at low concentrations and under physiologically stressful conditions [29]. Dhabhar and Viswanathan [30] showed that 50 ng/ml CORT was equivalent to the concentration of that produced after physiological stress, which had a stimulating immunological effect on macrophages and helped mice to adapt quickly to acute stress. To elucidate the role of CORT in modulating inflammatory response, we pretreated macrophages with different concentrations of CORT (0–900 ng/ml), which simulates the in vivo role of CORT after surgical trauma. In this study, lower concentrations of CORT (≤300 ng/ml) increased NLRP3 expression, indicating that low concentrations of natural glucocorticoids may exert proinflammatory effects on macrophages. These results are consistent with previous observations demonstrating that glucocorticoids can affect innate immunity by enhancing the expression of TLR2 [31]. Following trauma and surgery, the HPA axis is activated due to the stress response, resulting in the release of large quantities of glucocorticoids from the adrenal cortex into circulation, and glucocorticoids increase by more than 10-fold after stress. However, higher concentrations of CORT (≥700 ng/ml) inhibited LPS-induced NLRP3 expression, showing that CORT may exert an anti-inflammatory effect through the modulation of NLRP3 expression. Additionally, CORT also modulated cleaved caspase-1 expression in a similar manner. Therefore, it can be concluded that as an endogenous glucocorticoid, CORT modulates NLRP3 expression and its inflammasome priming, regulating innate immunity through the NLRP3 inflammasome.

These findings have important implications for the understanding of immunoregulation by endogenous glucocorticoids. They suggest that endogenous glucocorticoids released at the early stage of acute or moderate stress enhance immune function, which might help prime the innate immune system to defend against pathogens or injury. Consistent with an early report, in the central nervous system, glucocorticoids have been shown to be proinflammatory, increasing the levels of IL-1*β* and TNF-*α* in microglial cells [32]. Moreover, dexamethasone can directly enhance NLRP3 expression and activity in cultured and primary macrophages, which, in turn, leads to enhanced NLRP3 inflammasome activation [33]. Following trauma or surgery, excessive release of glucocorticoids exerts anti-inflammatory effects through downregulating NLRP3 expression, which might be of great importance in preventing subsequent pathogen invasion or tissue injury from an overactivated systemic inflammatory response. In addition to the concentrations of glucocorticoids, different cell types, experimental conditions, and binding of endogenous and synthetic glucocorticoids to GR may contribute to different research results. Additionally, these findings help to explain why moderate doses of glucocorticoids are still recommended during the perioperative period because of the regulatory effect on inflammation [34].

The exact mechanism of NLRP3 inflammasome activation is unclear, and extensive research has shown that various intracellular triggers that activate the NLRP3 inflammasome can also produce ROS. ROS production is closely related to mitochondria, and intracellular ROS mainly originate from mitochondria. Thus, mitochondria and their ROS act as a common signal mediating NLRP3 inflammasome activation [13]. However, Bauernfeind et al. [35] demonstrated that ROS inhibitors dose-dependently inhibited the expression of NLRP3, leading to blocking NLRP3 inflammasome activation. The promoter region of NLRP3 contains binding sites for AP-1, Sp1, Ets, and Myb, and ROS enhances their expression and leads to the transcriptional regulation of NLRP3 [36]. XO is an enzyme mainly localized to the mitochondrial compartment and mediates mitochondrial ROS generation. In addition to the anti-inflammatory effect of CORT in inhibiting NLRP3 expression, the present results demonstrated for the first time that higher concentrations (greater than 700 ng/ml) of CORT also downregulated XO expression and activity. Moreover, allopurinol, an inhibitor of XO, reduced NLRP3 expression. The present data suggests that XO regulates LPS-induced NLRP3 expression.

GR is the main effector molecule through which glucocorticoids exert their actions and is expressed ubiquitously in the cytoplasm of many cells. The present results demonstrated that addition of the GR antagonist RU486 increased NLRP3 and XO expression and XO activity. Based on the present data, it can be concluded that higher concentrations of CORT inhibit LPS-induced NLRP3 expression by suppressing XO activity mainly via GR. Finally, similar to the NLRP3 expression results, supernatant IL-1*β* and Il-18 levels were measured and confirmed that CORT inhibits NLRP3 inflammasome priming and final activation via suppressing XO.

Although we found that XO, which modulates ROS, is involved in modulating NLRP3 expression, we did not measure intracellular ROS, specifically mitochondrial ROS production. Whether ROS can regulate inflammasome assembly directly and by other mechanisms associated with inflammasome activation, in addition to modulating NLRP3 expression via XO, remains unknown. Finally, in vivo studies are needed to confirm the above results.

In conclusion, we have shown that LPS induces NLRP3 expression in macrophages at different time points, higher concentrations of CORT downregulate NLRP3 expression, and lower concentrations of CORT upregulate NLRP3. NLRP3 expression determines the activity of the NLRP3 inflammasome, and the NLRP3 inflammasome is crucial in recognizing invasive microbial pathogens and endogenous damage molecules and in triggering a cascade of inflammatory responses as a first line of defense. XO is closely related to NLRP3 inflammasome priming. CORT modulates the inflammatory response through the regulation of NLRP3 expression and inhibits NLRP3 inflammasome priming and activation via suppression of XO activity. Thus, innate immune responses are compromised, which may be a host defense mechanism to avoid excessive inflammatory responses and maintain balance but may also increase the possibility of infection after surgical stress. Therefore, the present study provides new insights into the modulation of innate immunity and the inflammatory response after surgical insults. Although additional studies are required to fully understand the modulation of NLRP3 expression and NLRP3 inflammasome activity by XO and its potential molecular mechanisms, our study describes for the first time that higher concentrations of endogenous CORT are involved in the downregulation of NLRP3 expression and its inflammasome activity in macrophages in vitro and provides a potential target, such as XO, for the modulation of inflammation after surgical injury and in NLRP3-associated diseases, such as autoinflammatory diseases, gout, and diabetes.

## Data Availability

The data used to support the findings of this study are included with the article.

## Supplementary Materials

Supplementary Materials Fig. S1: expression of NLRP3 mRNA in RAW264.7 cells treated with LPS (1 *μ*g/ml) for different times. In the control group, the cells were stimulated with LPS at 0 h. Values are mean (s.d.) (*n* = 5). ∗*P* < 0.05, ∗∗*P* < 0.01 versus control. Fig. S2: NLRP3 mRNA level in RAW264.7 cells treated with CORT and LPS. Cells were pretreated with 700 ng/ml CORT for 1 h and stimulated with LPS (1 *μ*g/ml) for different times. In the control group, there were no treatments. In the CORT group, cells were pretreated with 700 ng/ml CORT for 1 h and stimulated with LPS at 0 h. Values are mean (s.d.) (*n* = 6). ∗*P* < 0.05, ∗∗*P* < 0.01 versus the CORT group. Fig. S3: XO mRNA levels in RAW264.7 cells treated with CORT and LPS. Fig. S4: NLRP3 mRNA levels in RAW264.7 cells treated with allopurinol. Cells were pretreated with 250 *μ*g/ml of allopurinol for 1 h and stimulated with LPS (1 *μ*g/ml) for different times. In the control group, there was no treatment. In the LPS group, the time point for stimulation with LPS was 2 h with no allopurinol pretreatment. Values are mean (s.d.) (*n* = 6). ∗*P* < 0.05, ∗∗*P* < 0.01 versus the LPS group.
