# Supplementary material for: Corticosterone Inhibits LPS-Induced NLRP3 Inflammasome Priming in Macrophages by Suppressing Xanthine Oxidase
Source: Mediators Inflamm. 2020 May 18;2020:6959741. doi: 10.1155/2020/6959741 (PMC7251469; doi:10.1155/2020/6959741)
Supplement: Supplementary Materials — Fig. S1: expression of NLRP3 mRNA in RAW264.7 cells treated with LPS (1 μg/ml) for different times. In the control group, the cells were stimulated with LPS at 0 h. Values are mean (s.d.) (n = 5). ∗P < 0.05, ∗∗P < 0.01 versus control. Fig. S2: NLRP3 mRNA level in RAW264.7 cells treated with CORT and LPS. Cells were pretreated with 700 ng/ml CORT for 1 h and stimulated with LPS (1 μg/ml) for different times. In the control group, there were no treatments. In the CORT group, cells were pretreated with 700 ng/ml CORT for 1 h and stimulated with LPS at 0 h. Values are mean (s.d.) (n = 6). ∗P < 0.05, ∗∗P < 0.01 versus the CORT group. Fig. S3: XO mRNA levels in RAW264.7 cells treated with CORT and LPS. Fig. S4: NLRP3 mRNA levels in RAW264.7 cells treated with allopurinol. Cells were pretreated with 250 μg/ml of allopurinol for 1 h and stimulated with LPS (1 μg/ml) for different times. In the control group, there was no treatment. In the LPS group, the time point for stimulation with LPS was 2 h with no allopurinol pretreatment. Values are mean (s.d.) (n = 6). ∗P < 0.05, ∗∗P < 0.01 versus the LPS group. [file 6959741.f1.pdf]

Corticosterone inhibits LPS-induced NLRP3 inflammasome priming in macrophages by suppressing xanthine oxidase

Supporting information

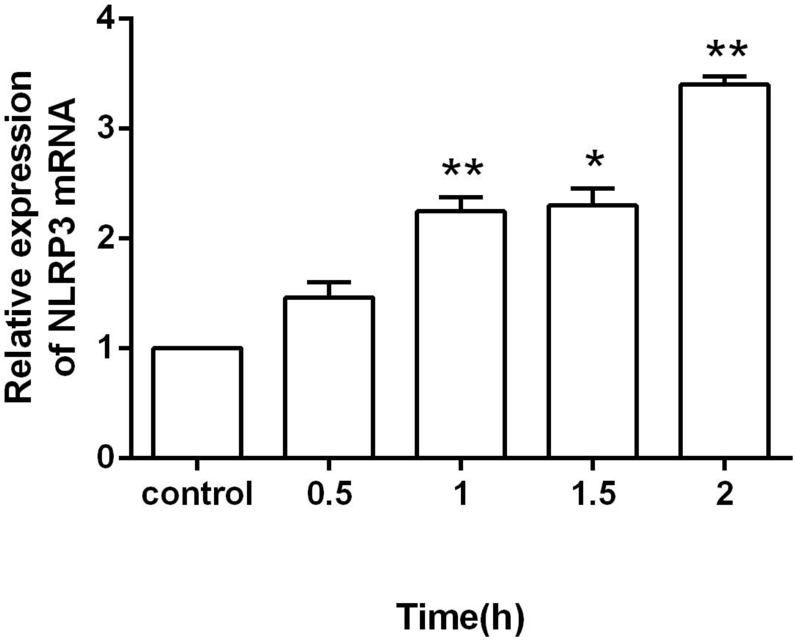

Fig. S1 Expression of NLRP3 mRNA in RAW264.7 cells treated with LPS (1 μg/ml) for different times. In the control group the cells were stimulated with LPS at o h. Value are mean (s.d.) (n=5). * P<0.05, ** P<0.01 *versus* control.

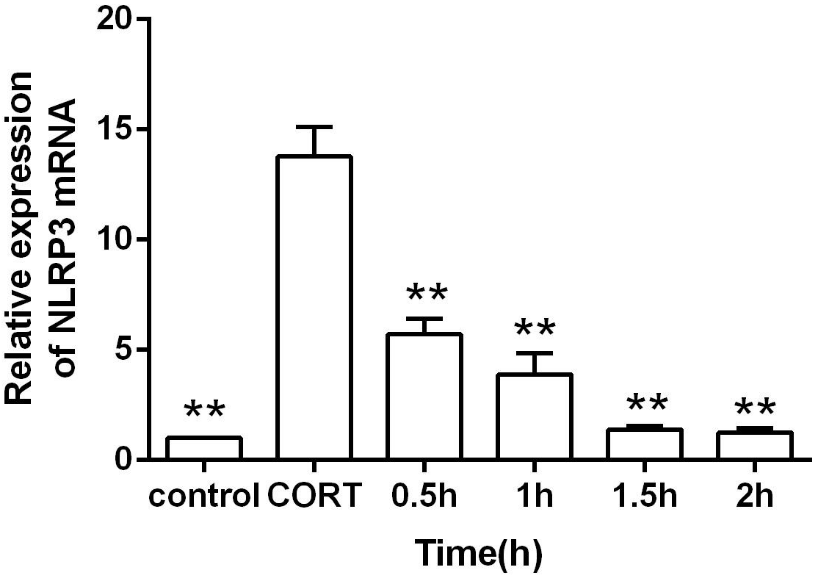

Fig. S2 NLRP3 mRNA level in RAW264.7 cells treated with CORT and LPS. Cells were pretreated with 700 ng/ml CORT for 1 h and stimulated with LPS (1 μg/ml) for different times. In the control group there were no treatments. In the CORT group, cells were pretreated with 700 ng/ml CORT for 1 h and stimulated with LPS at 0 h. Value are mean (s.d.) (n=6). * P<0.05, ** P<0.01 *versus* CORT group.

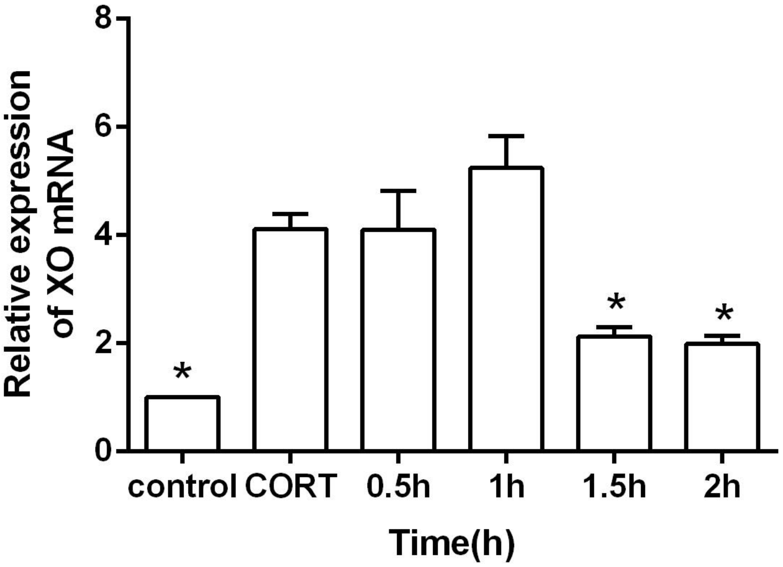

Fig. S3 XO mRNA levels in RAW264.7 cells treated with CORT and LPS.

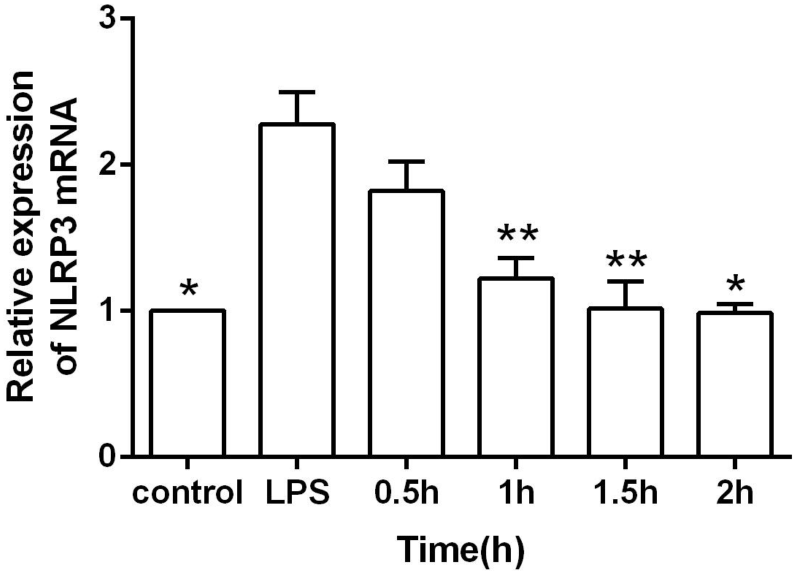

Fig. S4 NLRP3 mRNA levels in RAW264.7 cells treated with allopurinol. Cell were pretreated with 250 μg/ml of allopurinol for 1 h and stimulated with LPS(1 μg/ml) for different times. In the control group there were no treatment. In the LPS group, the time point for stimulation with LPS was 2h with no allopurinol pretreatment. Values are mean(s.d.) (n=6). * P<0.05, ** P<0.01 *versus* LPS group.
